# The Application of FT-IR Spectroscopy for Quality Control of Flours Obtained from Polish Producers

**DOI:** 10.1155/2017/4315678

**Published:** 2017-01-22

**Authors:** Katarzyna Sujka, Piotr Koczoń, Alicja Ceglińska, Magdalena Reder, Hanna Ciemniewska-Żytkiewicz

**Affiliations:** ^1^Department of Chemistry, Faculty of Food Sciences, Warsaw University of Life Sciences, Nowoursynowska 159 C, 02-787 Warsaw, Poland; ^2^Department of Food Technology, Faculty of Food Sciences, Warsaw University of Life Sciences, Nowoursynowska 159 C, 02-787 Warsaw, Poland

## Abstract

Samples of wheat, spelt, rye, and triticale flours produced by different Polish mills were studied by both classic chemical methods and FT-IR MIR spectroscopy. An attempt was made to statistically correlate FT-IR spectral data with reference data with regard to content of various components, for example, proteins, fats, ash, and fatty acids as well as properties such as moisture, falling number, and energetic value. This correlation resulted in calibrated and validated statistical models for versatile evaluation of unknown flour samples. The calibration data set was used to construct calibration models with use of the CSR and the PLS with the leave one-out, cross-validation techniques. The calibrated models were validated with a validation data set. The results obtained confirmed that application of statistical models based on MIR spectral data is a robust, accurate, precise, rapid, inexpensive, and convenient methodology for determination of flour characteristics, as well as for detection of content of selected flour ingredients. The obtained models' characteristics were as follows: *R*^2^ = 0.97, PRESS = 2.14; *R*^2^ = 0.96, PRESS = 0.69; *R*^2^ = 0.95, PRESS = 1.27; *R*^2^ = 0.94, PRESS = 0.76, for content of proteins, lipids, ash, and moisture level, respectively. Best results of CSR models were obtained for protein, ash, and crude fat (*R*^2^ = 0.86; 0.82; and 0.78, resp.).

## 1. Introduction

Flour, a product obtained in the process of grain milling, is one of the major raw materials in food industry. Flour is indispensable for production of a wide variety of staple products such as bread, pasta, cakes, and biscuits. Flour must be of satisfactory quality for processing to obtain acceptable end products.

Basic technological parameters of flour are content and quality of proteins, content of ash, amylolytic activity expressed as falling number or moisture. Lipids, another flour component, is also important, especially due to calorific value of flour. Gluten protein in flour creates a three-dimensional structure during dough mixing. Flour therefore must contain adequate amount of quality protein, which will hold bloated starch granules and gas bubbles within the dough structure. Bread and yeast cakes demand flour of high protein content, while other cake products can be made with flour of low protein content. Ash content does not really influence usable properties of flour; it is, however, the basic parameter for flour classification. Moisture values inform us indirectly about flour storage conditions. Excessive moisture facilitates growing of moulds; inadequate moisture, under 12%, promotes rancidity in fats present in flour [1]. The falling number determines the ability of dough to start and maintain fermentation process. Dough made with flour with a high falling number grows slowly and resulting bread is often of low-brown crust. Flour characterized by a low falling number usually causes viscous crumb in bread [2]. Lipids interact hydrophobically with nonpolar amino acids and participate an important role in creation the gluten complex. Free fatty acids form as a results of lipases action get oxidised and products formed enforce gluten structure [1].

In view of the considerable variety in grain composition, production of flour with correct and consistent quality parameters is a challenge and requires continuous monitoring of flour parameters during the production process. Classical analytical methods applied in laboratory are usually time-consuming, laborious, and impossible to be applied inline or online. Fourier transform infrared spectroscopy (FT-IR), on the other hand, is a fast, environmentally friendly, repeatable, and simple technique, which can be readily applied for flour quality control and thus help ensure proper quality of the final product. With FT-IR, especially when used with a Horizontal Attenuated Total Reflectance (HATR) accessory, there is no need for sample pretreatment [3, 4]. FT-IR spectroscopy offers qualitative and quantitative determination of food macrocomponents such as proteins [5, 6], lipids [7, 8], saccharides [9, 10], and water [11, 12]. FT-IR spectroscopy, which relies on absorption of infrared radiation by oscillating molecules, is increasingly applied in food research, like cakes and flakes [13], flours [14], nuts [15, 16], oils [17], meat [18, 19], and spirit beverages [20, 21]. Research tends to concentrate mainly on nonbread cereals like corn, rice, oats, and products obtained with them [22–26]. Investigations, in most cases, utilize near infrared range (NIR) [27–30]. In this study, an attempt was made to correlate spectral data of middle infrared range (MIR) with the analytical data of classical reference methods. The aim was to construct robust statistical models allowing differentiation of various flours due to of content of proteins, lipids (including fatty acids composition), water, and ash as well as evaluate amolytical properties and calorific value of unknown flour samples. The levels of protein fat and ash content were also measured and compared.

## 2. Materials and Methods

### 2.1. Materials

Eleven flour types obtained from four different grains, wheat (type 550, 650, 2000, and 2000 eco), rye (720, 1400, and 2000), spelt (1100 and 2000), and triticale (700 and 2000), as well as spelt bran have been investigated. According to Polish standards flours are classified by the content of ash. Type of four is expressed as percentage amount of ash of dry matter of a given flour. Details on studied types of flour types are presented in Table 1. Triticale flour was obtained in laboratory by milling grain from “Strzelce” company (Borowo, Poland), with a Quadrumat Senior mill (Brabender). Wheat, rye, and spelt flours came from different Polish mills producing flours for bakeries and retailers. Rye flours and wheat 550 flour were produced by Jelonki Ltd. (Ostrów Mazowiecka, Poland). Wheat flours types 650 and 2000, spelt flour, and bran were produced by Młyny Wodne Ltd. (Korczew, Poland).

### 2.2. Reference Procedures

Protein content, lipids content, ash content, falling number, and moisture were determined according to the ISO and Polish standards: PN-A-04018:1975, PN-A-74039:1964, ISO 2171:1994, ISO 3093:2007, and ISO 712:2009, respectively. Calorific value and fatty acid composition were determined according to the method described further. Three replicates were conducted in each case. In all above measurement, except fatty acid content measured twice.

#### 2.2.1. Protein Content

Protein content was determined indirectly by measuring the total nitrogen content. A flour sample (0.5 g) was mineralized for 30–60 minutes with 12 mL of 35% sulphuric acid (VI) and catalyst (3.5 g K_2_SO_4_ and 0.4 g CuSO_4_·5H_2_O). The Labtec™ Line Digestion Systems (FOSS, Hillerød, Denmark) was used. Then 80 mL of water and 20 mL of 40% sodium hydroxide solution were added and entire mixture was placed in Kjeltec system 1002 distilling Unit (FOSS, Hillerød, Denmark) for 4 min. 25 mL of 4% boric acid was added to distillate obtained and titrated by 0.1 mol L^−1^ hydrochloric acid against Tashiro indicator [31]. Nitrogen content was calculated as follows:

(1) $$\begin{array}{c} \%\;N=V_{\text{HCl}}*0.0014*200, \end{array}$$

*V*_HCl_ is the difference of volume of HCl used for sample and control trial [mL], 0.0014 is content of nitrogen [g] corresponding to 1 mL of 0.1 mol L^−1^ HCl solution, 200 is dilution factor.

The nitrogen-to-protein conversion factor was % *N∗*6.25 for rye flours and % *N∗*5.7 for others flours.

#### 2.2.2. Crude Fat Content

A 5.0 g of flour sample was predried in an air-drier at 105°C to obtain constant weight and then placed in a Soxtec 2055 System (FOSS, Hillerød, Denmark) to extract lipids with petroleum ether. Extraction was performed during 120 minutes at 155°C and proceeded in four steps: boiling, rinsing, solvent recovery, and drying. Crude fat content was calculated as follows [32]:

(2) $$\begin{array}{c} \frac{m_{2}-m_{1}}{m_{2}}*100, \end{array}$$

*m*_1_ is mass of sample after extraction [g], *m*_2_ is mass of sample before extraction [g].

#### 2.2.3. Ash Content

Ash content was determined by weighting remnants of the burning process of a 5.0 g flour sample conducted at 900°C for 1 hour in muffle furnace (FCF 73, Czylok Company) [33].

#### 2.2.4. Falling Number

Falling number was measured according to the Hagberg-Perten method [34] by measuring time of falling of a stirrer placed in a test-tube containing heated flour suspension. The 25 mL distilled water was added to the sample of flour shaken and placed in instrument. To keep constant proportion between water content and dry mater the amount of flour used was calculated based on its moisture; for example, 7 g of flour with 15% content of water was used [34]. The temperature of heating was 100°C.

#### 2.2.5. Moisture

Moisture was measured by the weighting method according to the Polish standard [35]. A 10 g sample was heated and dried in an air-drier at 130°C for 1.5 hours. Moisture was determined as a difference in the weight of the sample before and after heating.

#### 2.2.6. Caloricity

Calorific value was measured with a KL-12Mn calorimeter (Precyzja Bit Company). A flour sample pellet of an exact weight was placed in a fixed-volume bomb and burnt in a pressurized environment high in pure oxygen. Based on the temperature increase, the sample mass, and the bomb constant, the heat of combustion was calculated as follows:

(3) $$\begin{array}{c} Q=\frac{K*\left(T_{3}-T_{2}-k\right)}{m}, \end{array}$$

where *K* is calorimetric constant [J/°C], *T*_2_, *T*_3_ are temperatures of heating balance [°C], *k* is correction for the calorimeter-environment heat exchange, *m* is sample mass [g].

*k* was calculated as follows:

(4) k=0.5∗0.2∗T2−T1+0.2∗T4−T3+0.2∗n−1∗T4−T3,

where *n* is time of the main period in minutes (time of the increase of temperature from *T*_2_ to *T*_3_), *T*_1_, *T*_4_ are characteristic temperatures of heating balance.

#### 2.2.7. Fatty Acids Composition

Fatty acids content was determined by gas chromatography with a Shimadzu model GC-17A gas chromatograph equipped with a flame-ionization detector and a 30-metre capillary column of 0.22 mm, that is, with a film thickness of 0.25 *μ*m. The column temperature was programmed to increase from 60 to 230°C and the injector and detector ports were set at 225°C and 250°C, respectively. Detailed procedure is presented in Reder et al. [13].

### 2.3. FT-IR Spectroscopy

The 2000 System Perkin Elmer instrument operated by PEGRAMS software running on Windows 95 platform was used to register FT-IR spectra. The transmission technique was applied to conduct 25 scans for each of the studied flours in the spectral range of 4000–370 cm^−1^. KBr matrix pellets were prepared by mixing 300 mg of KBr with 1 mg of sample in laboratory ball mill. Then mixture was pressed in laboratory press with press 10 tones. Ready pellet was placed in measuring holder-dedicated accessory of System 2000 spectrometer and placed in measuring chamber. Average spectrum was considered final. The resolution was 4 cm^−1^ and the shift velocity 2 cm s^−1^. DTGS (deuterated triglycine sulphate) detector is a part of used spectrometer.

### 2.4. Statistics and Modeling

Statistical procedures were carried out using Statgraphics Plus 5.1 software. Statistically significant differences between flours were calculated with the one-way ANOVA method (Tukey's procedure). Spectral data, for example, integral intensity of 12 selected bands, were correlated with the content of the selected flour ingredient.

TQ Analyst running on Windows XP platform was used to search for the best statistical models correlating the spectral and the chemical data. The cross-validation diagnostics with one-left-out procedure was used to validate the models. The spectra were automatically normalized and mean-centered. Bond frequencies and intensities were used to calibrate statistical models. Maximal number of 20 factors was set for the tested models. For all parameters identical spectral pretreatment was applied.

## 3. Results and Discussion

In Poland, chemical and physical composition of flours is regulated by the two Polish standards [36, 37] for wheat and rye flour, respectively. Content of certain chemicals in flour depends mainly on grain variety and milling technology. The content of components analyzed in this study is presented in Tables 2 and 3. As data shows, the studied flours differ considerably with regard to their content of various chemicals.

### 3.1. Chemical Composition of Analyzed Flours

#### 3.1.1. Protein

Among the studied flours, the highest content of protein was observed for spelt flour. Its value increased with the flour type and ranged from 12.07 to 12.61%. The lowest protein content was in rye flour. This is obviously related to lower protein content in the rye grain than in the wheat and spelt grains. Protein content of 13.39% in bran was higher than those detected in flours. Statistically significant differences (*p* < 0.05 at confidence level 95%) in protein content were observed for all groups analyzed flours.

#### 3.1.2. Crude Fat

The content of crude fat varied between 1.5 and 3.5%. The highest level of crude fat was determined in bran. The lowest level was observed in wheat flour type 550 and 650 as well as triticale flour type 700. Differences between lower types (550, 650, 700, and 720) and higher types (1100, 1400, and 2000) of flours were statistically significant. The higher flour type the higher content of crude fat. This is related to distribution of lipids in the grain. The higher content of lipid is characteristic for the germ and the aleurone layer. As low type flour is made of material containing endosperm, the lipid level is at its lowest. On the other hand, bran which is made of mostly of the external part of the grain contains more lipids.

#### 3.1.3. Fatty Acids Composition

Table 3 contains qualitative and quantitative data on fatty acid composition of fats in the studied samples. The level of linoleic acid, one of the most unsaturated fatty acids, was the highest among the detected acids (54.58–61.36%). The level of two other unsaturated fatty acids, oleic acid and linolenic acid, in the studied flours was marked. Unsaturated fatty acids made up over 80% of the fatty acids total in the flours. Only three saturated fatty acids were identified: palmitic, stearic, and arachidic. The highest value of palmitic acid (23.59%) was determined in wheat flour type 650, while the lowest (15.99%) in rye flour type 720. The levels of stearic and arachidic acids were the lowest among the detected saturated fatty acids.

#### 3.1.4. Ash

According to the standard [33], ash is a noncombustible remnant obtained after oven incineration at 900°C. Measurement of ash content is commonly used in the milling industry worldwide as an indicator for bran contamination or flour purity. Mineral elements are present mainly in the external parts of grain, so that a higher content of bran reflects in a higher content of minerals.

The studied flours differed in ash content. The high content of ash was characteristic for bran while wheat flours of lowest types, 550 and 650, characterize the lowest content of this parameter. Overall, the level of ash was the highest in spelt flour which suggests the highest content of mineral elements in the spelt grain compared to the wheat, rye, and triticale grain. Differences between spelt, wheat, rye, and triticale flour type 2000 were statistically significant. Wheat flour type 2000 eco originated from ecological source had statistically significant higher content of ash as compared to the wheat flour same type originated from the nonecological source.

#### 3.1.5. Falling Number

The falling number values varied between 107 and 317 seconds. The highest value was observed for spelt flour, while the lowest for rye flour. Optimal falling number was observed for wheat flours: 243 and 248 for 2000 type and 242 and 261 for 550 and 650 types, respectively. The highest amylolitical activity was observed in the cases of rye and triticale flours which confirmed previous literature data: Makowska and Stachowiak [38] and Słowik et al. [39]. Differences in falling number between wheat flour types 550, 2000, and 2000 eco types as well as between spelt flours were statistically insignificant.

#### 3.1.6. Moisture

Moisture percentages of the analyzed flours varied between 11.1 and 13.7%. The highest value was observed for wheat flour type 550. The Polish standard allows for the moisture percentage below 15%. In the case of bran, moisture level should not exceed 11% [40].

#### 3.1.7. Calorific Value

The method of determination of calorific value relies on temperature measurement during the isochoric transformation. The deciding components for the calorific value of the studied flours were ordered as follows: lipids, proteins, and carbohydrates with energy equivalents of 39.55 J/g; 26.65 J/g; and 17.37 J/g, respectively. Therefore, flours with the highest content of lipids and proteins should have the highest calorific value which was confirmed by the measurements. The spelt products had the maximum calorific value. For wheat, rye, and triticale flours an increase in combustion value was observed along with the increase in the type of flour. The calorific values of the studied flours were not poles apart, although some marked differences were registered. The lowest value (15477 J/g) was obtained for triticale flour type 700, while the highest (16778 J/g) for spelt flour type 1100.

### 3.2. Spectral Analysis and Modeling

The experimental determination of protein, fat, ash, and fatty acids contents as well as properties such as moisture, the falling number, and energetic value in flour is laborious and time-consuming. The literature contains a lot of examples of correlation of various characteristics with IR spectra in different foodstuffs [41–43]. High correlation coefficients, simplicity of sample preparation, no need for any chemicals during measurement, short time of experiment, and robust results are only some of the advantages of application of the spectral methods instead of the traditional ones [3, 4]. Another strong advantage of FT-IR is that more than one parameter can be determined based on data of just a single spectrum [25]. Time needed for spectrum registration is quite short, and once registered, spectral data are simply entered into a preconstructed statistical model. One of the disadvantages is narrow application of a given model only for a set of similar products [3, 4].

**Figure 1:**
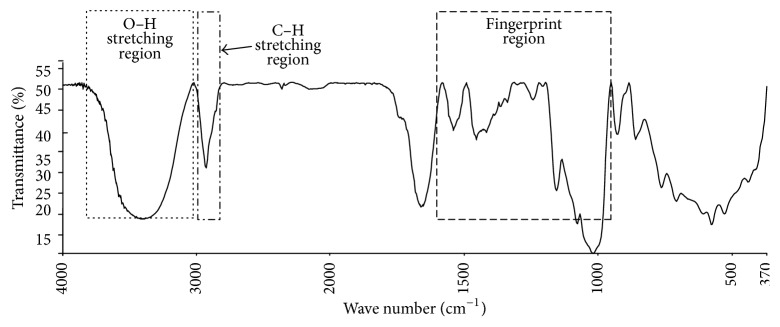
Spectrum of spelt flour type 1100.

The spectral data were correlated with the reference results in two different ways. The classical square regression (CSR) and partially least square (PLS) techniques were applied. The spectral ranges used for those analyses are presented in Table 4. Several bands characteristic for proteins, fats, carbohydrates, and water were observed in flours spectra. Intense band in the range 3600–3200 cm^−1^ is generated by stretching vibration of O–H bond. Bands in the range 3000–2800 cm^−1^ are assigned to stretching vibrations of C–H bond. Spectral region between 1500 and 900 cm^−1^ is called fingerprint region because of the unique patterns characteristic for given sample. The assignment of spectral bands to vibrations generating these bands is presented on Figure 1.

#### 3.2.1. Classic Square Regression

The linear regression analysis presents the relation between two variables: the ratio of two selected spectral bands (an dependent variable) and the content of a given chemical (independent variable). Coefficients of determination (*R*^2^) were used as a statistical measure of the model fitting.

The classical regression analysis was applied to correlate data on chemical composition and spectral data, where spectral data were expressed as ratio of intensity of two different bands. Content of protein, fat, ash, and linolenic acid and values of the falling number were correlated with the spectral data at statistically significant level. Correlations of some spectral data with some other flour features, for example, water content, caloricity, and remaining fatty acids, were of relatively small determination coefficients.  Table 5 presents selected statistical data, for example, coefficients of determination or linear function formulas. The highest determination coefficients, that is, 0.86, was obtained for protein, the second highest, that is, 0.83 for ash, and third highest, that is, 0.78 for crude fat content. For protein, the highest coefficients of determination were obtained for the ratio of two bands: one in the spectral range of 1583–1494 cm^−1^, which is defined in literature as amide band II, and a band in the spectral range 952–886 cm^−1^. For lipids, the highest coefficients of determination were obtained for the ratio of the bands located in the spectral ranges of 1197–952 cm^−1^ and 952–886 cm^−1^, respectively. For the linolenic acid content correlation calculated was the lowest, and three coefficients of determination were below 0.80 (see Table 5). Correlation coefficients calculated for calorific value were statistically insignificant.

#### 3.2.2. Partial Least Square Models

The data obtained in this study allowed the construction of four different statistical models using the PLS technique. The obtained correlations (statistical models) may have wide practical applications, at the very least in the preliminary assessment of technological value of flour. Remaining flour parameters, listed in chapter, were also tested to find robust models correlating spectral data with given feature. Unfortunately, statistically significant models were not obtained.

The samples in the calibration/validation set were of slightly different chemical compositions due to different grain sources and milling processes. Those differences were both quantitative and qualitative in nature because the spectral variations observed manifested them as increase/decrease in absorbance in a given spectral range [44].

Optimal calibration equations were explored using the PLS-1 regression. Optimal number of factors in constructing a model is very important since adding too many (more factors) which might come from noise in the data (the so-called overfit model) may decrease prediction strength of a model. On the other hand, if too few factors are used (the so-called underfit model), prediction accuracy for unknown samples will suffer as not enough factors are used to express all spectral variations influencing a given value. Therefore, it is very important to define a model that consists of the right number of factors to model a given value properly [44]. The optimal number of PLS factors to include in the calibration was evaluated by comparing determination coefficients (*R*^2^) between the actual and predicted values (those included in the calibration set), the Root Mean Square Error of Calibration (RMSEC), the Root Mean Square Error of Prediction (RMSEP), and the Prediction Residual Error Sum of Squares (PRESS) values. The higher *R*^2^ and the lower RMSEC, RMSEP, and PRESS, the more precise the model. Those statistical parameters have been used previously for different foodstuffs analyses [18, 44, 45].

The models of minimal RMPSEC, RMSEP, and PRESS and maximal *R*^2^ values with a given number of factors numbered I, II, III, and IV were calculated for proteins, lipids, ash, and moisture level, respectively. The calculated models' parameters were as follows: *R*^2^ = 0.97, PRESS = 2.14, RMSEC = 0.22, RMSEP = 0.32, and factors number = 10; *R*^2^ = 0.96, PRESS = 0.69, RMSEC = 0.11, RMSEP = 0.13, and factors number = 3; *R*^2^ = 0.95, PRESS = 1.27, RMSEC = 0.38, RMSEP = 0.55, and factors number = 2; and *R*^2^ = 0.94, PRESS = 0.76, RMSEC = 0.11, RMSEP = 0.15, and factors number = 5, for models I, II, III, and IV, respectively. Statistical parameters calculated for every model are presented in Table 6.

Spectral ranges for all models were assigned based on initial visual inspection which resulted in detection of most distinct differences in spectra. For model I the following spectral regions were selected: 3025–2800 cm^−1^; 1834–1583 cm^−1^; 1583–1494 cm^−1^ and 1494–1280 cm^−1^. Within the regions selected, there were 497 spectral data points.

For model II the following spectral regions were selected: 3846–3027 cm^−1^; 1834–1583 cm^−1^; 1279–1221 cm^−1^; and 952–886 cm^−1^. Within the regions selected, there were 1194 spectral data points.

For model III the following spectral regions were selected: 1279–1221 cm^−1^; 1197–952 cm^−1^; 952–886 cm^−1^. Within the regions selected, there were 369 spectral data points.

For model IV the following spectral regions were selected: 3846–3027 cm^−1^ and 1279–1221 cm^−1^. Within the regions selected, there were 877 spectral data points.

To construct, validate, and test each model, the total number of 60 samples (5 repetitions for each of 11 flour types and 1 bran) was randomly divided into two groups of 48 and 12 elements, separately for each measured parameter. Each of the 48-element group was used for model calibration and validation. The 12-element groups, containing precisely one sample of each studied flour type and bran, were treated as unknown independent samples not included in the model calibration and used to test the obtained models. It meant that those flour samples could be any samples purchased in any shop at any time.

The values of the studied parameters (proteins, lipids, ash, and moisture) of these samples were predicted with the created model while their actual values were measured by standard methods. Subsequently, the two sets of values were correlated. There was a linear correlation between the actual and predicted values of the studied parameters [44].

**Figure 2:**
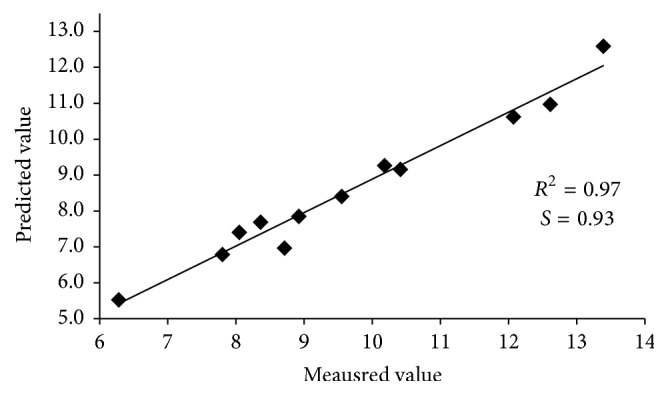
The linear correlation between the actual and the model-predicted protein level values in 12 flours samples of different origin.

Figure 2 presents a sample linear correlation between the actual and predicted protein level values of the 12 samples not included in calibration or validation of the model. There is a very good conformity between the two data sets with correlation coefficient, *R*^2^ = 0.97 and slope, *S* = 0.93. The parameters of the linear correlation between the model-predicted and actual values are as follows: *R*^2^ = 0.96, *S* = 0.91; *R*^2^ = 0.95, *S* = 0.93; *R*^2^ = 0.94, *S* = 0.91 for lipids, ash, and moisture, respectively.

## 4. Conclusions

(1) CSR procedure produced statistically significant relationship for selected bands intensities ratio and level of protein, lipids, ash, linolenic acid, and falling number.
(2) Four statistically significant models calibrated with use of PLS were obtained for content of proteins, lipids, and ash well as moisture level. Those models can serve to studied flours. Models parameters are as follows:
  Model I (protein content): *R*^2^ = 0.97, PRESS = 2.14, and factors number = 10.
  Model II (lipids content): *R*^2^ = 0.96, PRESS = 0.69, and factors number = 3.
  Model III (ash content): *R*^2^ = 0.95, PRESS = 1.27, and factors number = 2.
  Model IV (moisture level): *R*^2^ = 0.94, PRESS = 0.76, and factors number = 5.
(3) Interestingly, in the case of selected parameters, for example, linolenic acid, the simpler CSR procedure produced better results than the more sophisticated PLS technique.
(4) In the case of calorific value no statistically significant correlations with spectral data were determined.
(5) Compering the results obtained by meaning of two different statistical techniques one can conclude (see Tables 5 and 6) that PLS process slightly better.

## Figures and Tables

**Table 1:** Details of studied flour types.

Type of flour	Ash content
550	0.51–0.58%
650	0.59–0.60%
700/720	0.59–0.78%
1100	0.79–1.20%
1400	1.31–1.60%
2000	≤2%

**Table 2:** Chemical composition of the analyzed flours.

Flour	Protein [%]	Moisture [%]	Falling number [s]	Ash [%]	Crude fat [%]	Calorific value [J/g]
Wheat, type 550	7.80 ± 0.06^b^	13.7 ± 0.06^h^	261 ± 0.71^f^	0.49 ± 0.01^a^	1.5 ± 0.06^a^	15613.67 ± 255.24^a^
Wheat, type 650	10.18 ± 0.14^g^	12.6 ± 0.07^ef^	242 ± 0.71^g^	0.55 ± 0.01^b^	1.7 ± 0.08^b^	15723.64 ± 186.26^ab^
Wheat, type 2000	9.55 ± 0.17^f^	11.7 ± 0.3^abc^	243 ± 2.12^f^	1.36 ± 0.01^e^	2.4 ± 0.04^e^	16229.83 ± 225.16^bc^
Wheat, type 2000 eco	8.71 ± 0.05^d^	12.4 ± 0.05^de^	248 ± 1.41^f^	1.46 ± 0.02^f^	2.3 ± 0.09^de^	15734.50 ± 186.01^ab^
Spelt, type 1100	12.07 ± 0.15^i^	11.9 ± 0.27^bcd^	317 ± 2.12^h^	0.93 ± 0.03^d^	2.0 ± 0.07^c^	16778.33 ± 149.91^d^
Spelt, type 2000	12.61 ± 0.13^j^	12.2 ± 0.09^cde^	316 ± 2.83^h^	1.85 ± 0.02^h^	2.7 ± 0.03^f^	16498.50 ± 320.75^cd^
Spelt bran	13.39 ± 0.05^k^	11.4 ± 0.30^ab^	—	5.58 ± 0.03^i^	3.5 ± 0.05^g^	16685.33 ± 165.38^cd^
Rye, type 720	6.28 ± 0.15^a^	11.1 ± 0.39^a^	158 ± 2.83^b^	0.69 ± 0.00^c^	1.9 ± 0.02^b^	15589.50 ± 266.03^a^
Rye, type 1400	8.05 ± 0.09^bc^	12.7 ± 0.03^ef^	174 ± 2.83^c^	1.39 ± 0.02^ef^	2.2 ± 0.03^d^	15801.67 ± 371.64^ab^
Rye, type 2000	8.36 ± 0.07^c^	12.4 ± 0.08^de^	107 ± 2.12^a^	1.51 ± 0.02^g^	2.3 ± 0.06^de^	15840.33 ± 340.50^ab^
Triticale, type 700	8.92 ± 0.14^e^	13.4 ± 0.14^gh^	209 ± 2.12^e^	0.57 ± 0.01^b^	1.8 ± 0.02^b^	15477.83 ± 349.63^a^
Triticale, type 2000	10.41 ± 0.05^h^	13.0 ± 0.14^fg^	195 ± 2.12^d^	1.56 ± 0.01^g^	2.3 ± 0.02^de^	15874.00 ± 369.62^ab^

Data expressed as means ± standard deviation (*n* = 3). The different lower case letters (a–k) in the same column indicate significantly different values (*p* < 0.05); 11 homogenous groups are indicated with lower case letters a to k; the presence of the given letter after result includes this result into given group; for example, third column first raw, third raw, and forth raw represent homogenous group.

Honestly Significant Difference (HSD): protein, 0.34, moisture, 0.59, falling number, 8.41, ash, 0.06, lipid, 0.16, and calorific value, 543,99.

**Table 3:** Fatty acid composition (%) of crude fat extracted from the studied flours (*n* = 2).

Flour	Saturated fatty acids			Unsaturated fatty acids		
	Palmitic acid (C16:0)	Stearic acid (C18:0)	Arachidic acid (C20:0)	Oleic acid (C18:1) Cis	Linoleic acid (C18:2) cis	Linolenic acid (C18:3) cis
Wheat, type 550	18.17 (±0.02)	1.04 (±0.02)	0.58 (±0.03)	15.50 (±0.03)	61.00 (±0.02)	3.71 (±0.03)
Wheat, type 650	23.59 (±0.03)	0.83 (±0.02)	0.45 (±0.02)	13.35 (±0.02)	58.62 (±0.02)	3.17 (±0.03)
Wheat, type 2000	18.13 (±0.02)	0.79 (±0.03)	0.57 (±0.03)	15.21 (±0.03)	61.36 (±0.02)	3.94 (±0.02)
Wheat, type 2000 eco	17.21 (±0.02)	0.92 (±0.02)	0.83 (±0.03)	17.61 (±0.02)	59.23 (±0.02)	4.21 (±0.02)
Spelt, type 1100	22.51 (±0.02)	0.87 (±0.02)	0.62 (±0.02)	17.42 (±0.03)	55.38 (±0.03)	3.20 (±0.02)
Spelt, type 2000	16.14 (±0.03)	1.01 (±0.02)	0.87 (±0.03)	20.10 (±0.02)	58.32 (±0.03)	3.56 (±0.03)
Spelt bran	16.12 (±0.03)	1.04 (±0.02)	0.68 (±0.02)	21.50 (±0.02)	57.15 (±0.02)	3.51 (±0.02)
Rye, type 720	15.99 (±0.03)	0.58 (±0.02)	1.35 (±0.03)	18.05 (±0.03)	56.09 (±0.02)	7.93 (±0.02)
Rye, type 1400	19.23 (±0.02)	0.36 (±0.03)	0.82 (±0.03)	16.48 (±0.03)	55.02 (±0.03)	8.10 (±0.03)
Rye, type 2000	19.41 (±0.03)	0.52 (±0.02)	1.00 (±0.02)	17.34 (±0.03)	54.58 (±0.03)	7.15 (±0.02)
Triticale, type 700	20.09 (±0.02)	0.60 (±0.03)	0.57 (±0.03)	12.51 (±0.03)	60.98 (±0.03)	5.26 (±0.03)
Triticale, type 2000	22.91 (±0.03)	0.61 (±0.03)	0.47 (±0.03)	14.74 (±0.02)	55.43 (±0.03)	5.85 (±0.02)

**Table 4:** Spectral ranges used for CSR and PLS techniques.

Number of band	I1	I2	I3	I4	I5	I6	I7	I8	I9	I10	I11	I12
Spectral range [cm^−1^]	3846–3027	3025–2800	2389–2349	2349–2316	2287–2001	1834–1583	1583–1494	1494–1280	1279–1221	1197–952	952–886	885.5–370

**Table 5:** Data of CSR correlation analysis.

Parameter	Spectral bands used	Linear function formula	*r*	*R* ^2^
Protein	I7/I11	*y* = 4.36386 + 5.00414*x*	0.93	0.86
Protein	I9/I11	*y* = 1.34638 + 30.9113*x*	0.82	0.68
Crude fat	I1/I11	*y* = 0.747934 + 0.0389009*x*	0.88	0.78
Crude fat	I9/I11	*y* = 0.219968 + 7.46852*x*	0.81	0.65
Crude fat	I10/I11	*y* = 0.702822 + 0.0883232*x*	0.85	0.72
Crude fat	I11/I12	*y* = 5.01435 − 81.6113*x*	−0.82	0.67
Ash	I1/I11	*y* = −2.40758 + 0.103234*x*	0.89	0.80
Ash	I5/I11	*y* = −4.24408 + 7.48047*x*	0.86	0.74
Ash	I10/I11	*y* = −2.69006 + 0.24392*x*	0.90	0.81
Ash	I11/I12	*y* = 9.63411 − 237.645*x*	−0.91	0.83
Moisture	I3/I4	*y* = 12.311 + 0.0117*x*	−0.62	0.38
Caloricity	I9/I11	*y* = 14235.1 + 6545.66*x*	0.83	0.69
Falling number	I2/I5	*y* = −5.89366 + 42.01*x*	0.84	0.70
Falling number	I3/I7	*y* = 308.239 − 880.648*x*	−0.81	0.66
Palmitic acid	I2/I9	*y* = 18.668 + 0.0703*x*	−0.66	0.44
Stearic acid	I7/I11	*y* = 1.0444 − 0.0431*x*	0.75	0.56
Arachidic acid	I5/I7	*y* = 0.6244 + 0.0169*x*	0.79	0.62
Oleic acid	I10/I11	*y* = 16.727 − 0.0118*x*	0.69	0.48
Linoleic acid	I3/I7	*y* = 60.208 + 0.3762*x*	−0.63	0.40
Linolenic acid	I5/I7	*y* = 1.52499 + 4.16488*x*	0.83	0.69
Linolenic acid	I7/I9	*y* = 11.6075 − 1.72821*x*	−0.81	0.65
Linolenic acid	I7/I12	*y* = 10.9047 − 172.348*x*	−0.83	0.70

**Table 6:** Parameters describing the calibrated models obtained with PLS.

Parameter	Spectral range	RMSEC	RMSEP	RMSECV	*R* ^2^ calibration	*R* ^2^ prediction	PRESS	Factors number	Mean	Range
Proteins (model I)	I2, I6, I7, I8	0.22	0.32	0.29	0.97	0.93	2.14	10	9.69	6.28–13.39
Crude fat (model II)	I1, I6, I9, I11	0.11	0.13	0.18	0.96	0.94	0.69	3	2.21	1.5–3.5
Ash (model III)	I1, I4, I5, I6	0.38	0.55	0.19	0.95	0.94	1.27	2	1.49	0.49–5.58
Moisture (model IV)	I1, I9	0.11	0.15	0.19	0.94	0.89	0.76	5	12.37	11.1–13.7
Caloricity	I1, I2, I3, I6, I7, I8, I9, I10	81.5	43.8	1.06	0.85	0.92	26.89	3	15987.26	15477.83–16778.33
Falling number	I2, I3, I5, I7, I8	10.8	18.2	0.84	0.95	0.96	21.23	4	224.54	107–316
Palmitic acid	I2, I5, I6, I9, I11	1.31	1.88	1.49	0.71	0.51	105.27	3	19.12	15.99–23.59
Stearic acid	I2, I5, I6, I9, I11	0.16	0.19	0.21	0.25	0.17	1.99	2	0.76	0.36–1.04
Arachidic acid	I2, I5, I6, I9, I11	0.05	0.06	0.06	0.76	0.75	0.13	1	0.73	0.45–1.35
Oleic acid	I2, I5, I6, I9, I11	1.66	2.04	2.54	0.66	0.54	304.32	3	16.65	13.35–21.50
Linoleic acid	I2, I5, I6, I9, I11	1.18	1.14	1.84	0.76	0.42	159.78	6	57.76	54.58–61.36
Linolenic acid	I5, I7, I9, I12	1.32	1.76	1.43	0.006	0.10	95.84	1	4.96	3.17–8.10
